# Dynamics of cell rounding during detachment

**DOI:** 10.1016/j.isci.2023.106696

**Published:** 2023-04-18

**Authors:** Agata Nyga, Katarzyna Plak, Martin Kräter, Marta Urbanska, Kyoohyun Kim, Jochen Guck, Buzz Baum

**Affiliations:** 1 Cell Biology, MRC Laboratory of Molecular Biology, Cambridge CB2 0QH, UK; 2 MRC Laboratory for Molecular Cell Biology, University College London, London WC1E 6BT, UK; 3 Biotechnology Center, Center for Molecular and Cellular Bioengineering, Technische Universität Dresden, 01307 Dresden, Germany; 4 Max Planck Institute for the Science of Light & Max-Planck-Zentrum für Physik und Medizin, 91058 Erlangen, Germany; 5 Institute for the Physics of Living Systems, University College London, London WC1E 6BT, UK

**Keywords:** Molecular biology, Cell biology, Developmental biology

## Abstract

Animal cells undergo repeated shape changes, for example by rounding up and respreading as they divide. Cell rounding can be also observed in interphase cells, for example when cancer cells switch from a mesenchymal to an ameboid mode of cell migration. Nevertheless, it remains unclear how interphase cells round up. In this article, we demonstrate that a partial loss of substrate adhesion triggers actomyosin-dependent cortical remodeling and ERM activation, which facilitates further adhesion loss causing cells to round. Although the path of rounding in this case superficially resembles mitotic rounding in involving ERM phosphorylation, retraction fiber formation, and cortical remodeling downstream of ROCK, it does not require Ect2. This work provides insights into the way partial loss of adhesion actives cortical remodeling to drive cell detachment from the substrate. This is important to consider when studying the mechanics of cells in suspension, for example using methods like real-time deformability cytometry (RT-DC).

## Highlights

• Interphase cells actively round up following partial de-adhesion from the substrate
• Actomyosin contractility drives both interphase and mitotic cell rounding
• Active ERM proteins are required for detachment-induced interphase cell rounding
• Ect2 is not required for interphase cell rounding

## Graphical abstract

**Figure:**
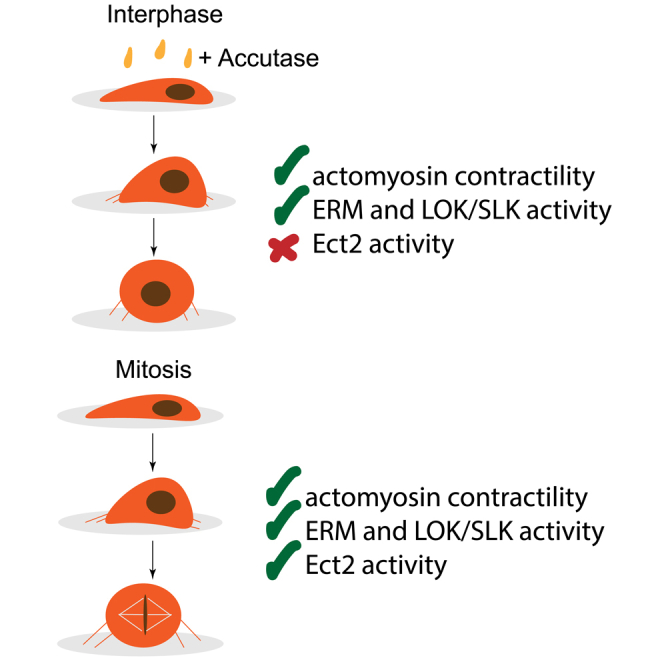

## Introduction

Once cells enter mitosis, they undergo a rapid change in shape. This occurs throughremodeling of integrin-based substrate adhesions^1^ an increase in cortical tension driven by the activation of Myosin II and ERM proteins,^2^^,^^3^ and an influx of water that leads to an increase in cell volume.^4^ Cells in interphase can also be seen rounding up. This can be an active process, e.g. when metastatic cancer cells de-adhere, enabling them to move through the vasculature or lymph.^5^ Alternatively, interphase cell rounding can be triggered by changes in the extracellular environment, e.g. following the loss of substrate and/or neighbors.^6^^,^^7^ Finally, cells also undergo this type of rounding whenever they are removed from the substrate during an experiment, e.g. during cell passaging or when lifting cells into supsension for analysis by real-time deformability cytometry (RT-DC)^8^ or cell sorting (FACS).

Although we have a relatively good understanding of the molecular and cellular events that accompany mitotic rounding, many of which occur directly downstream of Cdk1/CyclinB activation,^9^ it remains unclear how interphase cells round up. Nevertheless, there are likely to be close parallels between the two processes. Both involve a loss of substrate adhesion.^10^ Thus, mitotic cells remain flat when forced to adhere strongly to the substrate.^11^ In addition, just as mitotic cells stiffen as they round,^12^^,^^13^^,^^14^ interphase cells have been shown to be less compliant when detached and rounded, than when adhering to a substrate.^15^ These changes in cell mechanics likely reflect changes to the actomyosin cortex,^16^ whose organization is profoundly affected by mitotic entry, cell shape, external forces, and by both cell-cell and cell-substrate adhesion.

To better understand this process, in this paper we explore the molecular mechanisms that cause interphase cells to round up following their forced removal from the substrate. We show that a partial loss of substrate adhesion triggers actomyosin-dependent cortical remodeling and ERM activation, which facilitates further adhesion loss, as measured by Paxillin-GFP, causing cells to round as they enter suspension. Although the path of rounding in this case superficially resembles mitotic rounding in involving ERM phosphorylation, retraction fiber formation, and cortical remodeling downstream of ROCK, it does not require Ect2. Taken together this work provides insights into the way partial loss of adhesion actives cortical remodeling to drive cell detachment from the substrate.

## Results and discussion

### Modeling interphase rounding

To study the cell shape changes that follow the loss of cell-substrate adhesion, we analysed HeLa cells undergoing detachment induced by various treatments. Although Accutase, EDTA and Trypsin are all effective at inducing de-adhesion and rounding,^17^ to image the process live, we searched for a regime in which cell rounding and detachment were robust but occurred with kinetics slow enough to enable live cell imaging (ruling out Trypsin and Accutase at 37°C). This led us to focus our analysis on the effects of Accutase, an enzymatic treatment that induces the digestion of cell-matrix linkers without affecting cell viability or differentiation,^17^^,^^18^ at 22°C. This is a protocol often used to passage cells and/or when preparing cells for mechanical studies using RT-DC.^8^ Importantly, these Accutase-treated cells remained viable for long periods of time in suspension, and were able to respread on transfer onto a substrate (Figure S1), implying that the treatment does not remove all surface adhesion molecules.

### Kinetics of interphase and mitotic rounding

**Figure 1:**
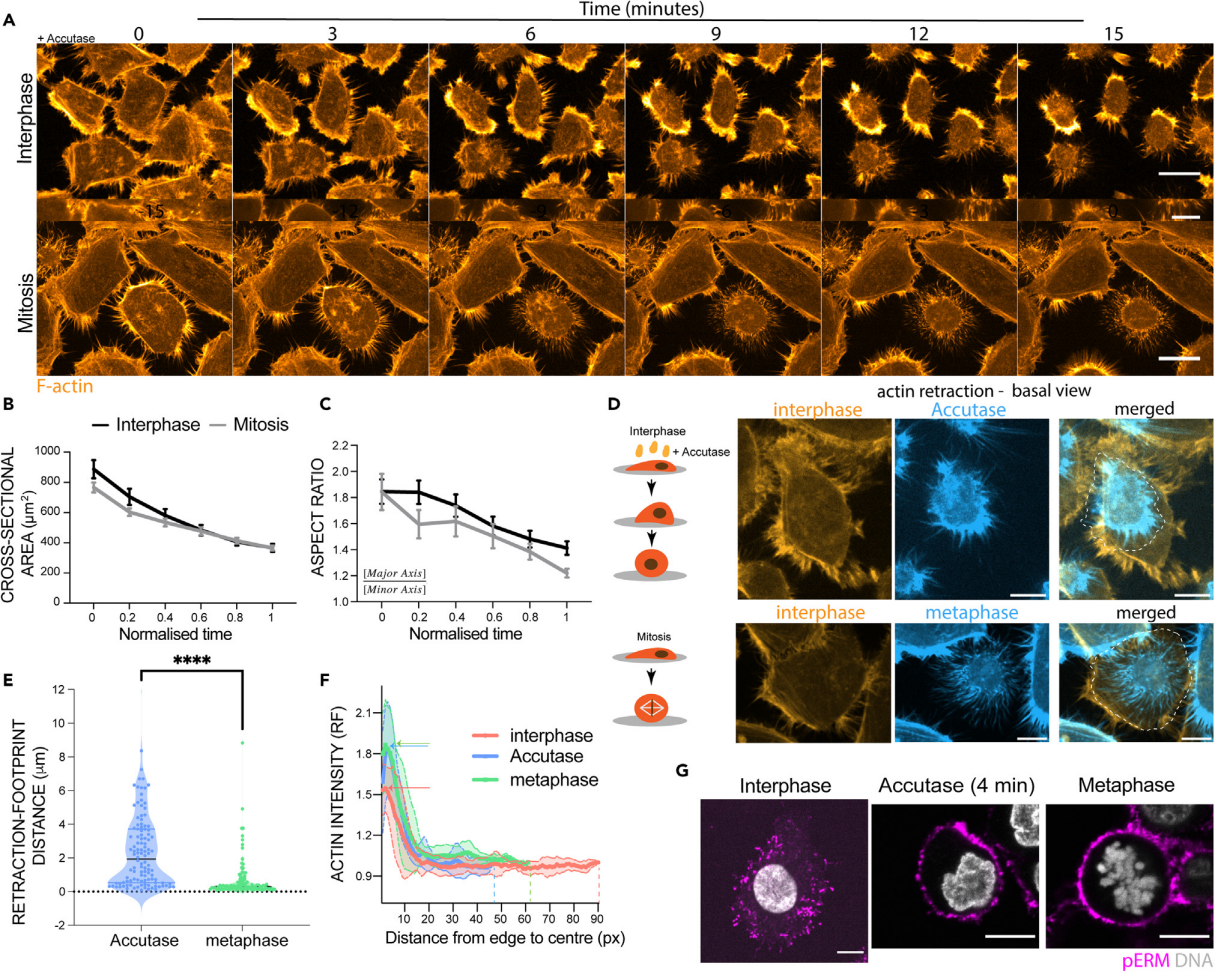
Shape changes during cell detachment from substrate (A) Time-lapse of HeLa cells treated with Accutase or during mitosis over 15 min at room temperature (RT). Orange – F-actin. Scale bar = 20 μm. (B) Cross-sectional area (μm^2^) of HeLa cells treated with Accutase (n = 25) or during mitosis (n = 13) over time (normalized with respect to start and end points). Datapoints represent mean ± SEM. Statistical difference: Šídák’s multiple comparisons test. (C) Aspect ratio (Major Axis/Minor Axis) of HeLa cells treated with Accutase (n = 25) or during mitosis (n = 13) over time (normalized with respect to start and endpoints). Datapoints represent mean ± SEM. Statistical difference: Šídák’s multiple comparisons test. (D) Graphical representation of cell detachment process following enzymatic detachment (Accutase) or during mitotic rounding. Basal footprint of interphase cells (orange) and detached cell (Accutase or metaphase in blue). Scale bar = 10 μm. (E) Distance (μm) between the edge of the interphase actin cortex and the limit of retraction fibers after rounding for cells entering mitosis and following treatment with Accutase (n = 100 retraction fibers from at least 5 different cells). Statistical difference, Mann-Whitney test, ∗∗∗∗p<0.001. (F) Actin intensity (relative fold change) from the cell edge (cortex) to the cell center of interphase cell, a spherical Accutase-rounded cell and a cell in metaphase (n = 5 independent cells). (G) Localization of actin-membrane linker pERM in adhered interphase cells, rounding up detaching interphase cells (4 min after Accutase treatment) and round mitotic cells. Magenta – pERM, Gray – DNA. Scale bar = 10 μm.

Mitotic rounding was shown previously to require a whole host of changes in cytoskeletal organisation and adhesion to round up. These are triggered downstream of CDK/CyclinB,^9^ and brought about via the activation of the RhoGEF, Ect2,^13^ and the inactivation of Rap1.^11^ To explore whether similar cellular and molecular mechanisms underlie cell rounding in interphase, we began by carrying out live imaging of HeLa cells fluorescently labeled for F-actin following treatment with Accutase at 22°C (Figures 1A and 1B). This treatment (Figure 1A) caused cells to undergo a gradual reduction in cross-sectional area (Figure 1B) and aspect ratio (Figure 1C) as they rounded up over a period of 15 minutes in a manner that superficially resembled cells entering mitosis.

Although both mitotic cells and cells treated with Accutase left retraction fibers behind as they rounded up, the details of the process differed in the two cases. Thus, although the tips of retraction fibers remained in place in rounding mitotic cells, likely as the result of mitotic-specific changes to integrin-based adhesions^1^, adhesions slipped in cells treated with Accutase (Figure 1E), as measured by the separation of the tips of retraction fibers from the adhesive interphase cell footprint (Figures 1D and 1E).

At the same time, we observed an increase in the intensity of actin filaments at the cortex in both Accutase-treated cells and mitotic cells (Figure 1F), as measured by changes in the localisation of LifeAct-GFP. In addition, like cells entering mitosis, cells treated with Accutase accumulated high levels of the active, phosphorylated form of the cortical membrane-actin linkers ezrin/radixin/moesin (ERM) as they rounded (Figure 1G). Thus, despite the subtle differences in adhesion remodeling in the two cases, cell rounding induced by forced partial de-adhesion of interphase cells from the substrate involves a series of changes to the cortex that resemble those induced upon entry into mitosis.

### pERM and ERM kinases LOK/SLK are required for interphase cell rounding

**Figure 2:**
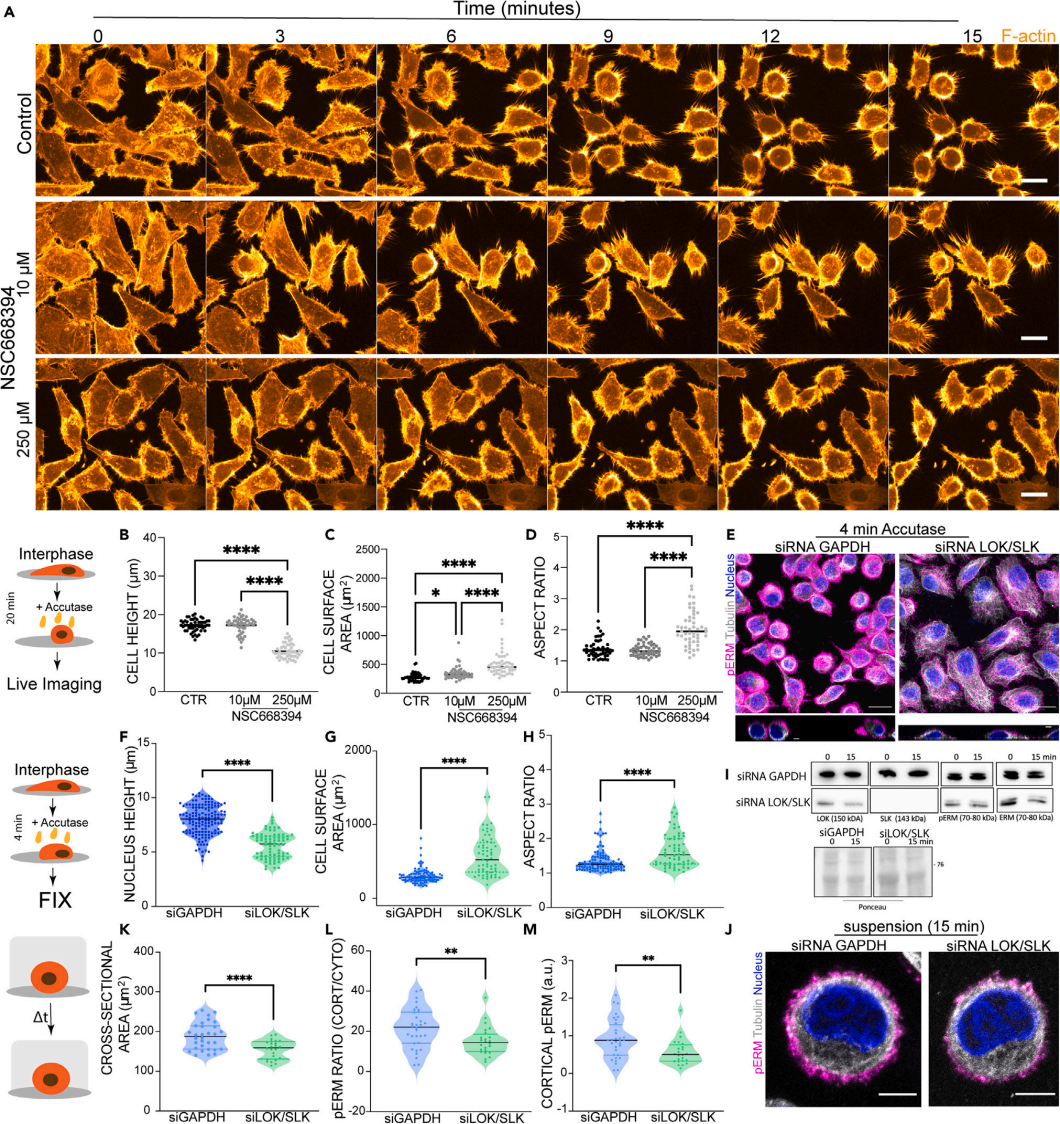
Role of ERM proteins and LOK/SLK kinases in shape regulation during cell detachment and in suspension (A) Time lapse of HeLa cells treated with Accutase over 15 min at RT following treatment with ERM inhibitor NSC668394 (10 μM overnight or 250 μM for 3 h). Orange – F-actin. Scale bar = 20 μm. (B–D) The shape changes of Control (n = 50) and NSC668394-treated (n = 50) HeLa cells following 20 min of Accutase treatment. Statistical difference, Kruskal-Wallis multiple comparison test, ∗p<0.05, ∗∗∗∗p<0.001. (B) Cell height (μm). (C) Cell surface area (μm^2^). (D) Aspect ratio for this experiment. (E) Immunofluorescence images of fixed HeLa cells treated with siRNA against GAPDH (Control) or combined LOK/SLK at 4 min after the Accutase treatment. Magenta – pERM, Gray - Tubulin, Blue – Nucleus. Scale bar = 20 μm. (F–H) Cell shape properties of HeLa cells treated with siRNA against GAPDH (as control) or combined LOK/SLK at 4 min after the Accutase treatment. Statistical difference, Mann-Whitney test, ∗∗∗∗p<0.001. F) Nucleus height (μm; GAPDH n = 147, LOK/SLK = 74), G) Cell surface area (μm^2^; GAPDH n = 100, LOK/SLK = 59), H) Aspect ratio (GAPDH n = 100, LOK/SLK = 59) for this experiment. (I) Western Blot showing Lok and Slk protein expression in HeLa cells immediately after detachment (0 min) and in suspension (15 min) following siRNA treatment against GAPDH (control), or following combined LOK and SLK RNAi. (J) Representative immunofluorescence images of GAPDH (Control) or combined LOK/SLK RNAi HeLa cells in suspension (15 min). Magenta – pERM, Gray - Tubulin, Blue – Nucleus. Scale bar = 5 μm. (K–M) Cell shape properties and pERM expression in HeLa cells previously treated with siRNAs against GAPDH (Control, n = 32) or both LOK/SLK (n = 24) after 15 min in suspension. Statistical difference, Mann-Whitney test, ∗∗p<0.01, ∗∗∗∗p<0.001. K) Cross-sectional area (μm^2^). (L) Ratio of the averaged cortical and cytoplasmic expression of pERM, M) Averaged intensity of cortical pERM for this experiment.

ERM proteins, such as Moesin in *Drosophila,* have previously been shown to be critical for mitotic rounding,^2^ whereas phosphomimetic ERM proteins can prevent cells spreading on the substrate.^19^ To investigate whether ERM proteins play a similar role in interphase cell detachment, we targeted ERM proteins with the inhibitor NSC668394, which prevents the T567 phosphorylation of ERM family members via direct binding.^20^ The addition of NSC668394 to cultures led to a significant impairment of detachment following Accutase treatment (Figure 2A) in a manner that depended on drug concentration. Thus, when exposed to low concentrations of NSC668394 (10 μM, overnight incubation), cells exhibited slightly reduced detachment dynamics following Accutase treatment, and were more spread than control cells after 20 minutes, the time by which control cells had assumed a spherical shape. Higher concentrations of NSC668394 (250 μM for 3 h) resulted in an even more profound rounding defect (Figures 2B–D).

Having shown that ERM proteins are activated upon rounding and that their inhibition with NSC668349 delays interphase rounding following Accutase treatment, we next investigated the upstream signaling cascades that might be responsible for ERM activation under these conditions, focusing on LOK (serine threonine kinase 10 STK10, lymphocyte oriented kinase LOK) and SLK (STE20-like kinase) kinases, which have been shown to phosphorylate ERM proteins in many systems.^21^^,^^22^

To silence the expression of both LOK and SLK, we combined siRNAs against the two kinases, and used siRNA against GAPDH as a control. The efficacy of the LOK/SLK siRNA effect was confirmed by western blotting. RNA-mediated gene silencing led to a significant loss of SLK protein, partial decrease in LOK protein, and was associated with a corresponding decrease in pERM levels (Figure 2I). This silencing of LOK and SLK was also found to impair both mitotic (FiguresS2A and S2B) and interphase rounding following a 4 minute treatment with Accutase (Figures 2E–2H). After 15 minutes, however, LOK/SLK siRNA treated cells had assumed a spherical shape, resembling that of GAPDH siRNA control cells (Figure 2J). This indicates that, once detached from the substrate, pERM proteins are not required for interphase cells to assume a completely spherical shape (although these cells appeared slightly smaller than controls (Figure 2K)). This was the case even though LOK/SLK siRNA cells in suspension had a markedly reduced ratio of cortical to cytoplasmic pERM (Figure 2L) and diminished levels of cortical pERM (Figure 2M). Taken together these data suggest that pERM levels become elevated as cells round in a manner that depends on LOK/SLK, but which is independent of the trigger (partial disassembly of adhesions or entry into mitosis^23^).

### Actin and ROCK are required for cell rounding during detachment

**Figure 3:**
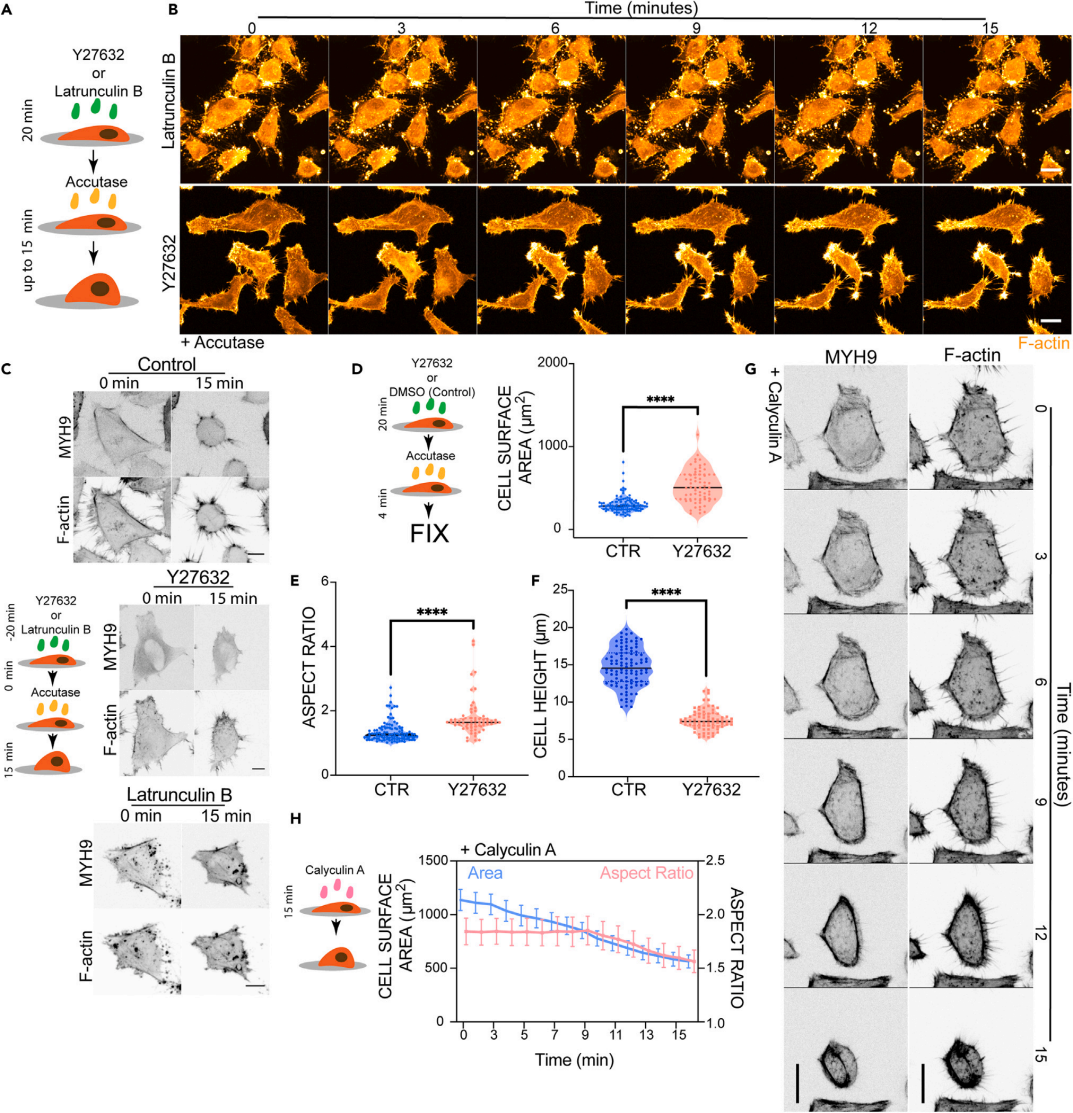
Role of actin and ROCK signaling in cell shape changes during detachment rounding (A) Graphical representation of a cell pre-treated with ROCK inhibitor (Y27632) or the actin inhibitor (Latrunculin B) being subjected to Accutase treatment. (B) Immunofluorescence images of representative Latrunculin B- or Y27632-pre-treated HeLa cells being induced to de-adhere from the substrate by Accutase treatment. F-actin in orange, Scale bar = 20 μm. (C) Snap-shots of control HeLa cells, and cells pre-treated with either Y27632 or Latrunculin B, at 0 and 15 min after Accutase treatment. Top panel, MYH9 (myosin II) expression. Bottom panel, F-actin expression. Scale bar = 10 μm. (D–F) The shape of control and Y27632-treated HeLa cells following 4 min of Accutase treatment. Statistical difference, Mann-Whitney test, ∗∗∗∗p<0.001. D) Cell surface area (μm^2^; CTR n = 100, Y27632 = 59), E) Aspect ratio (CTR n = 100, Y27632 = 59), and F) Cell height (μm; CTR n = 102, Y27632 = 74). (G) Time lapse of HeLa cells treated with Calyculin A (25 nM) over 15 min at RT. Right panel, F-actin in black. Left panel MYH9 in black. Scale bar = 20 μm. (H) Cell shape parameters of Calyculin A-treated (n = 25) HeLa cells over 15 min, including cross-sectional area (μm^2^, in blue) and aspect ratio (in pink).

Mitotic rounding also depends on actin and ROCK-mediated contractility.^24^ To test if the same is true during the interphase rounding induced by partial de-adhesion from the substrate, we pre-treated HeLa cells with the ROCK inhibitor Y27632 to decrease myosin activity, or with the actin monomer sequestering agent Latrunculin B (Lat-B) to depolymerize the actin cytoskeleton (Figure 3A). Twenty minutes later, cells were then treated with Accutase and imaged as they detached from the substrate (in the constant presence of inhibitors) (Figure 3B). In this experiment, the inhibition of ROCK using Y27632 (which decreased levels of cortical Myosin II, Figure 3C) led to a significant impairment in cell rounding relative to the corresponding control. As a result, Accutase-treated cells remained attached to spread on the substrate (Figures 3B and 3C). Disruption of the actin cytoskeleton using Lat-B reduced levels of both cortical actin and Myosin II and had a similar effect (Figures 3B and 3C). To better understand the impact of ROCK on cell rounding, we next measured cell shape changes in fixed Y27632-treated or DMSO-treated (control) cells exposed to Accutase for 4 minutes (Figures 3D–3F). Y27632-treated cells had a higher surface area (Figure 3D), a higher aspect ratio (Figure 3E), and a decreased cell height (Figure 3F). These data highlight the roles of both ROCK and actin in the process of interphase cell rounding up during detachment from the substrate.

Because both pERM proteins and active Myosin II have been reported to alter cell-substrate adhesion, it was important to determine whether or not Y27632-treated cells and NSC668349-treated cells remain flat and attached to the substrate after Accutase treatment as the result of increased interphase adhesion. To this end, we imaged Paxillin dynamics in control interphase cells and in cells treated with the two inhibitors before and after the addition of Accutase (Figure S3). Adhesions appeared similar in control and NSC668349-treated cells, but were markedly reduced in cells treated with Y27632, as shown previously.^25^ Thus, there was no correlation between the rate of rounding and the size of interphase adhesions as measured by Paxillin-GFP (Figure S3). In addition, adhesions in these experiments were seen disassembling as cells underwent rounding, but did not change in cells that remained flat. This implies that the cortical forces inducing rounding are important for adhesion disassembly in cells treated with Accutase. In line with this, when we induced the slow detachment of cells from the substrate using the calcium chelator EDTA instead of Accutase (FiguresS4A–S4E), we observed much slower loss of Paxillin-GFP adhesions. In this case, too, adhesion remodeling and rounding depended on ROCK. These data suggest that there is positive feedback in the system, as the loss of adhesions triggers cortical contractility, which further reduces cell-substrate adhesion. Note that although adhesions in Y27632 treated cells following the addition of Accutase were still present, they appeared visibly smaller than those in control cells. Thus, although cells remained flat and attached to the substrate long after Accutase addition, they could be detached using mechanical force (banging the plate).

To compare the cell biology of DMSO-treated and Y27632-treated cells detached using this shake-off method in detail, we incubated cells in suspension for different times (immediately, t = 0 min, or at t = 30 min), before fixing and staining them to monitor cell shape and cytoskeletal organization. The confocal microscopy analysis of these cells revealed that although DMSO-treated cells round up as they detach, it takes time for Y27632-treated cells to fully round following detachment (Figures S5A and S5B). After remaining in suspension for 30 min, however, both DMSO and Y27632 cells appear as near perfect spheres.

When we monitored the localization of the active phosphorylated ERM proteins in suspension HeLa cells +/− ROCK inhibitor, we observed relatively uniform and elevated phosphorylated ERM staining in DMSO-treated cells at early timepoints after the Accutase treatment. By contrast, the cortical recruitment of pERM proteins appeared incomplete in Y27632-pre-treated cells immediately after detachment from the substrate (Figure S5D). As they rounded, however, these cells accumulated relatively uniform and high levels of cortical pERM (FiguresS5C and S5D). Furthermore, the increase in cortical accumulation of pERM in these ROCK inhibitor-treated cells was found to correlate with cell shape as measured by cell aspect ratio (Figure S5E). These data imply that ERM activation is responsive to cell shape (as has been suggested previously^26^), and does not strictly require the activity of ROCK. Thus, although ROCK is required for cells to round following the loosening of their adhesions, and accelerates de-adhesion and cell rounding in suspension, ROCK is not required for detached cells to assume a final spherical shape – which represents a minimal energy conformation.

### Ect2 is not required for interphase cell rounding

**Figure 4:**
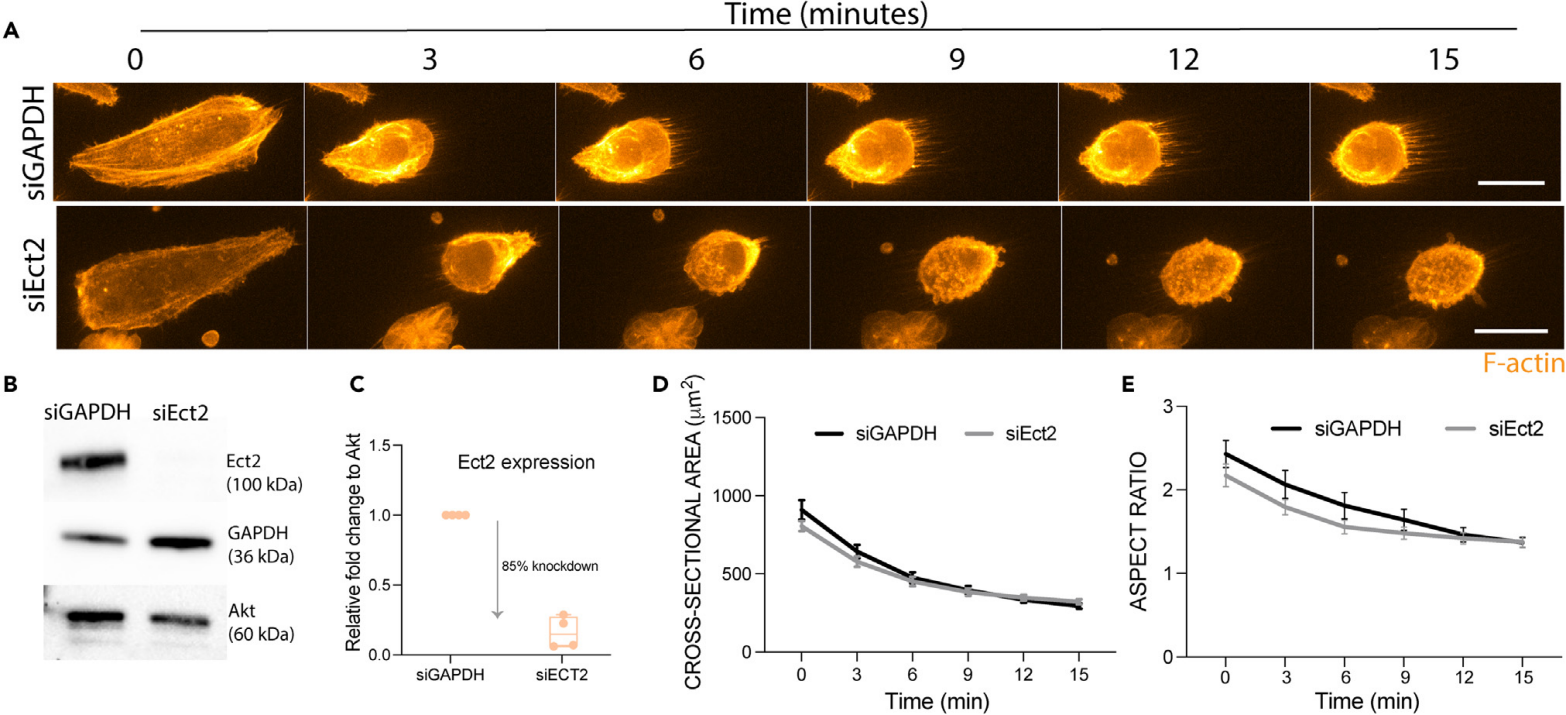
Ect2 is not required for interphase cell rounding during detachmen (A) Time lapse of HeLa cells treated with Accutase over 15 min at RT following treatment with siRNA against Ect2. Orange – F-actin. Scale bar = 20 μm. (B and C) Changes in Ect2 protein expression following 24 hours of siRNA treatment measured using B) Western Blot, and C) quantified against loading control of Akt protein (two separate experiments carried out in duplicates). (D and E) The shape changes of Control (n = 41) and Ect2 (n = 43) siRNA-treated HeLa cells following 20 min of Accutase treatment. Statistical difference: Šídák’s multiple comparisons test. D) Cell cross-sectional area (μm^2^). (E) Aspect ratio (Major Axis/Minor Axis).

The data presented thus far show that interphase cell rounding following Accutase treatment is driven by similar processes as mitotic rounding. A common trigger is the weakening of substrate adhesions, which occurs either through enzymatic digestion (Accutase), or by adhesion remodeling downstream of Cdk1/CyclinB activation (mitosis). In addition, rounding in both cases is promoted by the activation of the ROCK-Myosin II and SLK/LOK-dependent pERM activation. These similarities are surprising given the differences in the mechanical properties of the cortex in rounded interphase cells and mitotic cells.^27^ Given this, we thought it important to identify differences in the two modes of rounding. As shown previously, Ect2 knockdown impacts the rounding of mitotic cells.^13^ In our interphase experiments, Ect2 knockdown (Figures 4B and 4C) had no visible impact on the detachment of HeLa cells from surface following Accutase treatment (Figure 4A). Interphase Ect2 RNAi cells were of similar size and shape to control cells at the beginning of Accutase treatment, and decreased their surface area at the same rate as control cells while rounding up during detachment (Figures 4D and 4E). This is in line with the idea that Ect2 is largely inactive in interphase, where it is localized largely to nucleoli,^28^ and is first activated in the cytoplasm at G2/M. These data suggest that different cues can trigger the same type of cortical remodeling to drive cell rounding.

Finally, to determine if the phosphorylation-induced activation of Myosin II and ERM are sufficient to induce rounding, we performed the time-course experiments following the addition of Calyculin A to cell cultures (Figure 3G). Calyculin A is known to increase levels of activated Myosin,^29^^,^^30^ pERM^19^ and to reduce integrin-mediated cell adhesion.^19^ As observed previously with 3T3 fibroblasts^19^^,^^29^ and HEK293T cells,^19^ increasing cell contractility (via treatment with Calyculin A) was found to be enough to drive detachment, causing adherent HeLa cells to round up (Figures 3G and 3H).

### Conclusions

Taken together, these data suggest that cells entering mitosis and interphase cells rounding following de-adhesion follow a strikingly similar path of cell shape remodeling. In both cases, cells leave retraction fibers behind as they round. Moreover, rounding in the two cases is driven by a common process of actomyosin remodeling. In both cases, the trigger to round induces a similar upstream pathway (ROCK and LOK/SLK) leading to the activation of Myosin and pERM proteins respectively. In our data, the accumulation of pERM at the cortex was correlated with cell shape as measured with aspect ratio, and so may be a consequence as well as a cause of the rounding, providing positive feedback to the system. We speculate that the assembly of a rigid round cortex that is rich in actin and ERM proteins in both cases may be part of a general mechanism cells use to ensure that they are able to resist external forces and survive under crowded conditions (mitosis) and when subject to shear and flow (suspension, circulating metastatic cells).

The similarities between mitotic and interphase rounding are surprising given the differences in the reported cell biology of round interphase and mitotic cells, which include differences in actin cortex thickness,^3^ composition,^31^^,^^32^ filament organisation,^3^^,^^27^ stiffness,^12^^,^^13^^,^^14^ pressure^33^ and cytoplasmic viscosity.^34^ However, we also observed clear differences in specific aspects of the process of rounding in the two cases. First, the tips of the retraction fibers formed in Accutase treated cells were found to slide as cells rounded. By contrast, the tips of retraction fibers largely remained in place in cells entering mitosis, likely because of a specific program of mitotic cell adhesion remodeling,^1^ which is important to provide cells with a memory of the mother cell footprint as they divide. Second, interphase rounding induced by detachment from the substrate does not require Ect2 as an upstream trigger for ROCK activation. This makes sense, given the need for CDK1/CyclinB to trigger the nuclear export of Ect2 protein so that it can activate Rho and ROCK in the cytoplasm. This raises the question of nature of the trigger inducing activation of ERM proteins and Myosin II in interphase cells following their partial detachment from the substrate. Given the marked differences in the rates of actomyosin-dependent rounding triggered by Accutase and EDTA, we speculate that this may be a direct effect of the weakening of adhesions on signaling that impacts the actomyosin cortex. The disassembly of adhesions leads to activation of Myosin II, which in turn leads to a mechanically induced further loss of adhesions, and to rounding, which activates ERM proteins. Although a similar cascade may be triggered at mitotic entry, cells counter it by remodeling adhesions to ensure that some remain in place to guide respreading. In this way, our study helps to highlight the commonalities and important regulatory differences between these two types of rounding.

We note that although this analysis of interphase rounding involves studying the effects of an artificial treatment to induce the partial detachment of cells from the substrate, our findings are important to consider when interpreting experiments that involve the analysis of cells placed in suspension (e.g. RT-DC) – which as we show here have undergone profound and time-dependent changes in their cortical organization as they round both on the substrate and once they are in suspension. In addition, this work is likely to be relevant to the study of cancer cells which switch between adhesion-dependent and independent modes of motility as they undergo metastatic spread.

### Limitations of the study

There are several important limitations to our study. First, this study focused on the rounding following detachment in HeLa cells – a single cancer-derived cell line. The mechanisms described here might be different in normal epithelial cells or cells of different origin (e.g., mesenchymal). Second, by applying Accutase to cells at 22°C, we induced a relative slow cell detachment through partial de-adhesion from the substrate to better study the process in-depth without the induction of cell blebbing. Some of the observations made may depend on the precise kinetics of rounding. Third, in the future it will be important to test the relevance of the mechanisms of interphase cell detachment described here in a more physiological 3D environment, e.g., in organoids, spheroids or *in vivo* models.

## STAR★Methods

### Key resources table

**Table:** 

REAGENT or RESOURCE	SOURCE	IDENTIFIER
**Antibodies**		

anti-rabbit pERM	Cell Signalling Technology	Cat#3726S RRID:AB_10560513
anti-rabbit ERM	Cell Signalling Technology	Cat#3142S RRID:AB_2100313
anti-rabbit SLK	Abcam	Cat#ab65113 RRID:AB_1142917
anti-rabbit LOK	Bethyl Laboratories	Cat#A300-399A RRID:AB_386110
anti-rabbit Akt	Cell Signaling Technology	Cat#9272S RRID:AB_329827
anti-rabbit Ect2	Santa Cruz	Cat#sc-1005 RRID:AB_2246263
GAPDH-HRP	Thermo Fisher Scientific	Cat#MA515738HRP RRID:AB_2537659
Goat anti-rabbit HRP secondary antibody	Dako	Cat#P0448 RRID:AB_2617138
goat anti-rabbit Alexa Fluor 546 secondary antibody	ThermoFisher Scientific	Cat#A11035 RRID:AB_143051

**Chemicals, peptides, and recombinant proteins**		

Y27362	Sigma-Aldrich	Cat#Y0503
Calyculin A	Sigma-Aldrich	Cat#C5552
NSC668394	Merck Millipore	Cat#341216
Latrunculin B	Sigma-Aldrich	Cat#L5288
DMEM	Thermo Scientific	Cat#31966-021
foetal bovine serum (FBS)	Thermo Fisher Scientific	Cat#10270106
penicillin and streptomycin	Thermo Fisher Scientific	Cat#15070063
rat tail collagen type I	First Link UK Ltd.	Cat#60-30-810
Accutase	ThermoFisher Scientific	Cat#A1110501
Lipofectamine RNAiMax transfection reagent	ThermoFisher Scientific	Cat#13778075
Laemmli Buffer	Sigma-Aldrich	Cat#S3401
siRNA buffer	Dharmacon	Cat#B-002000-UB-100
MOPS buffer	ThermoFisher Scientific	Cat#NP0001
Luminata Crescendo Western HRP substrate	Merck Millipore	Cat#WBLUR0100
Trichloroacetic acid (TCA)	Sigma-Aldrich	Cat#T6399
Triton X-100	Sigma-Aldrich	Cat#T8787
Bovine serum albumin	Sigma-Aldrich	Cat#A7906

**Experimental models: Cell lines**		

Wild-type HeLa Kyoto	Mitocheck^35^	RRID:CVCL_1922
HeLa MYH9-GFP LifeAct-RFP	Lab of Prof. Ewa Paluch^3^	N/A
HeLa Paxillin-GFP LifeAct-RFP	Lab of Prof. Buzz Baum, made by Marina Fedrova, unpublished	N/A

**Recombinant DNA**		

siRNA Ect2 (FlexiTube siRNA, 20 nmol, Hs_ECT2_6)	Qiagen	Cat#1027418
siRNA GAPDH (ON-TARGETplus siRNA SMARTPools)	Dharmacon	Cat#L-004253-00-0005
siRNA SLK (ON-TARGETplus siRNA SMARTPools)	Dharmacon	Cat#L-003850-00-0005
siRNA LOK (STK10, ON-TARGETplus siRNA SMARTPools)	Dharmacon	Cat#L-004168-00-0005

**Software and algorithms**		

Fiji ImageJ	Schindelin et al.^36^	https://imagej.nih.gov/ij/
MATLAB (version R2020a)	Matlab	RRID:SCR_001622
GraphPad Prism 9	GraphPad	https://graphpad.com
Adobe Illustrator	Adobe	https://www.adobe.com/uk

### Resource availability

#### Lead contact

Further information and requests for resources and reagents should be directed to and will be fulfilled by the lead contact, Buzz Baum (bbaum@mrc-lmb.cam.ac.uk).

#### Materials availability

This study did not generate new unique reagents.

### Experimental model and subject details

#### Cell lines

Wild-type HeLa Kyoto^35^ (female, RRID:CVCL_1922), HeLa MYH9-GFP LifeAct-RFP^3^ and HeLa Paxillin-GFP LifeAct-RFP cells were cultured in DMEM (Cat#31966-021, Thermo Scientific) supplemented with 10% foetal bovine serum (FBS, Cat#10270106, Thermo Fisher Scientific) and 100 U/ml penicillin and 100 μg/ml streptomycin (Cat#15070063, Thermo Fisher Scientific) at 37°C with 5% CO_2_. HeLa cells were isolated from a female patient (ATCC). HeLa cell lines were tested regularly in-house for mycoplasma contamination and were not authenticated for this study.

### Method details

#### Perturbation experiments

To inhibit ROCK, cells were pre-treated for 20 min with Y27362 (Cat#Y0503, Sigma-Aldrich) at a final concentration of 10 μM. For suspension analysis, following detachment with Accutase (Cat#A1110501, ThermoFisher Scientific) for 5 min, cells were collected into complete DMEM (+/− Y27362), centrifuged at 500 xg for 5 min and resuspended in complete DMEM (+/− Y27362). To increase cell contractility, Calyculin A (Cat#C5552, Sigma-Aldrich) at final concentration of 25 nM was used and live imaging started immediately following its addition. Ezrin was inhibited with NSC668394 (Cat#341216, Merck Millipore) overnight at a final concentration of 10 μM or for 3 hr at 250 μM. For actin depolymerisation Latrunculin B (Cat#L5288, Sigma-Aldrich) was used at a final concentration of 20 nM. Equivalent volume of DMSO was used as controls. Accutase treatment and following incubation in suspension were performed at room temperature (22°C–25°C, RT). Accutase treatment had no effect on cell viability or ability to respread (Figure S1). Cells were also detached using EDTA (0.68M, in-house prepared solution).

#### Transfection

Cells were transfected using reverse transfection method. ON-TARGETplus siRNA SMARTPools were purchased from Dharmacon (Horizon Discovery) and diluted to 0.5 μM in 1x siRNA buffer (Cat#B-002000-UB-100, Dharmacon). Ect2 siRNA was purchased from Qiagen (FlexiTube siRNA, 20 nmol, Hs_ECT2_6, Cat#1027418) and diluted to 0.5 μM in RNAse-free water. Using 12-well cell culture dishes, 12 μl of GAPDH (Cat#L-004253-00-0005), 12 μl of SLK (Cat#L-003850-00-0005), 15 μl of LOK (STK10, Cat#L-004168-00-0005) siRNA or 5 μl Ect2 siRNA was added to 250 μl of OptiMEM (Cat#A4124801, ThermoFisher Scientific), while in a separate well 250 μl of OptiMEM was mixed with 2.5 μl of Lipofectamine RNAiMax transfection reagent (Cat#13778075, ThermoFisher Scientific). Solutions were incubated for 5 min at RT followed by addition of the 250 μl of OptiMEM+RNAiMax to the OptiMEM+siRNA solution and further incubation for 20 min at RT. In the meantime, HeLa cells were trypsinised and counted. After the incubation time, 40,000 cells per well were added to each well after the incubation time in 500 μl of DMEM with 10% FBS without antibiotics. Following 24 hr incubation at 37°C with 5% CO_2_, media was changed for complete DMEM. Cells treated with siRNA against Ect2 were analysed then and cells treated with siRNA against LOK and/or SLK were incubated for further 48 hr.

#### Western blotting

For western blotting, pellets of suspended cells were directly lysed in ice-cold 1X Laemmli Buffer (Cat#S3401, Sigma-Aldrich), sonicated for 1 min using Sonicator 3000 (Misonix), and boiled for 5 min at 95°C. Lysates were loaded onto NuPage 4–12% Bis-Tris gradient gels (Cat#NP0322BOX, ThermoFisher Scientific) with electrophoresis performed at 150V for 70 min in MOPS buffer (Cat#NP0001, ThermoFisher Scientific). Gels were transferred onto nitrocellulose membranes (Cat#15249794, Fisher Scientific) at 4°C and 100V for 60 min, followed by 30-minute blocking in 5% milk in TBST. Primary antibodies were diluted in 2.5% milk in TBST incubated overnight at 4°C on a rotor. Following antibodies were used: anti-rabbit pERM (1:2000, Cat#3726S, Cell Signalling Technology, RRID:AB_10560513), anti-rabbit ERM (1:2000, Cat#3142S, Cell Signalling Technology, RRID:AB_2100313), anti-rabbit SLK (1:1000, Cat#ab65113, Abcam, RRID:AB_1142917), anti-rabbit LOK (1:1000, Cat#A300-399A, Bethyl Laboratories, RRID:AB_386110), anti-rabbit Akt (1:500, Cat#9272S, Cell Signaling Technology, RRID:AB_329827), anti-rabbit Ect2 (1:500, Cat#sc-1005, Santa Cruz, RRID:AB_2246263), GAPDH-HRP (1:2000, Cat#MA515738HRP, Thermo Fisher Scientific, RRID:AB_2537659). Goat anti-rabbit HRP secondary antibody (Cat#P0448, Dako, RRID:AB_2617138) was used at 1:3000 for 2 hr at room temperature. The specific proteins were detected using a ChemiDoc MP Imaging System (BioRad) after incubation with Luminata Crescendo Western HRP substrate (WBLUR0100, Merck Millipore).

#### Live imaging

For live imaging of detaching cells, cells were plated on glass-bottom 4-well slides (μ-slides, ibidi GmbH, Thistle Scientific) or single glass-bottom dishes (ibidi GmbH, Thistle Scientific) coated with 100 μg/ml collagen type I (Cat#60-30-810, First Link UK Ltd.) 24 hours prior to imaging. Once the slides were inserted in the microscope holder, each well was imaged separately by firstly removing the cell culture medium, followed by addition of Accutase. Imaging started immediately after addition of Accutase using either a 40x/1.3NA oil objective mounted on an Andor Revolution Spinning Disk, 60x/1.2 NA water objective or SR HP Plan Apo Lambda S 100x/1.35 NA silicone immersion objective mounted on a W1 Spinning disk. The images of the cells were taken at 2 μm intervals with 5 slices in total at 3 min intervals (for a total of 21 min) at 25°C with 5% CO_2_. Following the live imaging acquisition, a full z-stack of the cells was taken at 500 nm intervals (a total of 30 μm thickness) for the quantification of cell shape properties after Accutase treatment. Fiji^36^ (version 2.3.0/1.53f, RRID:SCR_002285) was used for image analysis. The shape change during Accutase treatment was assessed through a maximal projection of cell images, and manual segmentation of cells together with measurement analysis using the “Analyse” shape plug-in. For the analysis of the cells at the end of treatment, the maximal projections were used to measure the cell surface area and aspect ratio, while the orthogonal view was used to manually measure the cell or nucleus height.

#### Immunofluorescence and confocal microscopy

To analyse cell shape change during Accutase treatment, cells were plated on collagen type I-coated glass-bottom 8-well slides (μ-slides, ibidi GmbH, Thistle Scientific) 24 hours before experiments. Cells were then treated with Accutase for 4 minutes and immediately fixed in ice-cold 10% trichloroacetic acid (TCA, Cat#T6399, Sigma-Aldrich) for 15 min. For studying cell shape after complete detachment in suspension, cells were plated in plastic 6-well plate dish 24 hours before experiments. Cells were then treated for 5 minutes with Accutase and the complete detachment from the dish was facilitate by a plate shake off. Cells were then resuspended in fresh media. Following either direct resuspension or further incubation in fresh media in suspension at room temperature (for 15 or 30 min), cells were centrifuged, supernatant aspirated and fixed in ice-cold 10% TCA for 15 min.

After fixation, both detaching and suspended cells were permeabilised for 5 min with 0.2% Triton X-100 (Cat#T8787, Sigma-Aldrich) in PBS and blocked with 5% bovine serum albumin (BSA, Cat#A7906, Sigma-Aldrich) in PBS for 30 min. Primary anti-rabbit pERM (Cat#3726S, Cell Signalling Technology, RRID:AB_10560513) antibody was diluted in 1% BSA/PBS at 1:500 dilution and incubated for 1 hr at RT, followed by incubation with goat anti-rabbit Alexa Fluor 546 secondary antibody (Cat#A11035, ThermoFisher Scientific, RRID:AB_143051) for 1 hr at RT. Samples were mounted with DAPI. Images were acquired using a Zeiss 780 (Zen software) confocal fluorescent microscope, with images taken using x63/1.4NA Oil objective of the equatorial plane of attached or suspended cells, with 15–30 z slices of 500 nm intervals. Fiji analysis software was used to analyse the images by subtracting background, performing maximal projection (of z planes of detaching cells, and the central planes for suspended cells (6 μm thickness)). Binary masks of cells were created using thresholding. In-house script was used in MATLAB (version R2020a, licencenumber 645993, RRID:SCR_001622) to provide automatic quantification of cell shape, and for suspended cells also cortical and cytoplasmic pERM intensities, their ratio, and cortical coverage.

### Quantification and statistical analysis

Each dot on the boxplots represents a measurement from a single cell. The experiments were performed in at least duplicates and repeated at least twice. Data are presented as means +/− SEM. For comparison of means between two categories Mann Whitney test was used. For comparison of means between multiple categories Kruskal-Wallis multiple comparison test was used. For comparison of multiple categories in different groups a 2-way ANOVA with Tukey’s multiple comparisons or Šídák’s multiple comparisons test was used. The difference was considered significant with p values of ∗p<0.05, ∗∗p<0.01, ∗∗∗p<0.005, and ∗∗∗∗p<0.001. All statistical analysis and plotting were carried out using GraphPad Prism (version 9.5.0, RRID:SCR_002798).

## Data Availability

• Microscopy data and western blot images reported in this paper will be shared by the lead contact upon request. • The paper does not report original code. • Any additional information required to reanalyze the data reported in this paper is available from the lead contact upon request.
