# Supplementary material for: Dynamics of cell rounding during detachment
Source: iScience. 2023 Apr 18;26(5):106696. doi: 10.1016/j.isci.2023.106696 (PMC10165398; doi:10.1016/j.isci.2023.106696)
Supplement: Document S1. Figures S1–S5 [file mmc1.pdf]

iScience, Volume *26*

Supplemental information

Dynamics of cell rounding during detachment

Agata Nyga, Katarzyna Plak, Martin Kräter, Marta Urbanska, Kyoohyun Kim, Jochen Guck, and Buzz Baum

Supplementary Figures

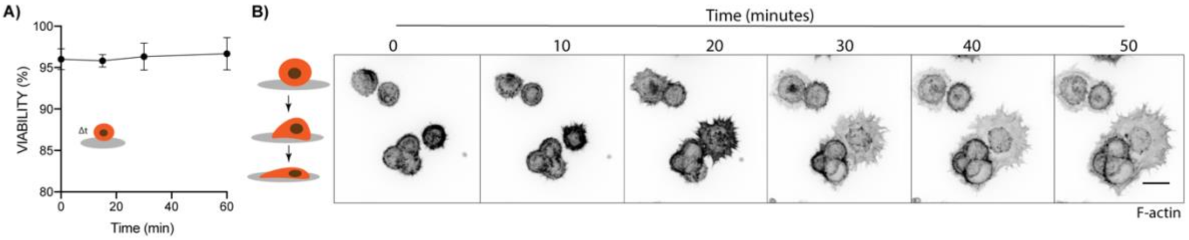

Supplementary Figure 1. Effect of suspension on HeLa cell viability and respreading dynamics – related to Figure 1 and Star Methods – Perturbation experiments.
A) Cell viability (%) of HeLa cells in suspension incubated at room temperature for up to 60 minutes. Viability measured with Trypan Blue (n=3); B) Time lapse of HeLa cells respreading on glass after incubation in suspension (50 min), F-actin in black, scale bar = 20 µm.

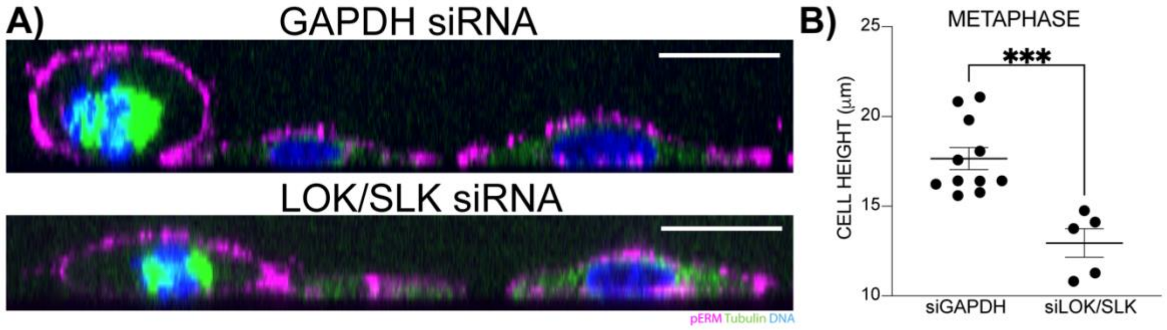

Supplementary Figure 2. Effect of LOK/SLK siRNA treatment on mitotic rounding – related to Figure 2.
A) Immunofluorescence images of GAPDH siRNA-treated (Control) or LOK/SLK siRNA-treated HeLa cells during mitotic rounding, Magenta – pERM, Green – Microtubules, Blue – Nucleus. Scale bar = 10 µm; B) Cell height measurements at metaphase of GAPDH and LOK/SLK siRNA-treated HeLa cells. Mann-Whitney test, ***p<0.005.

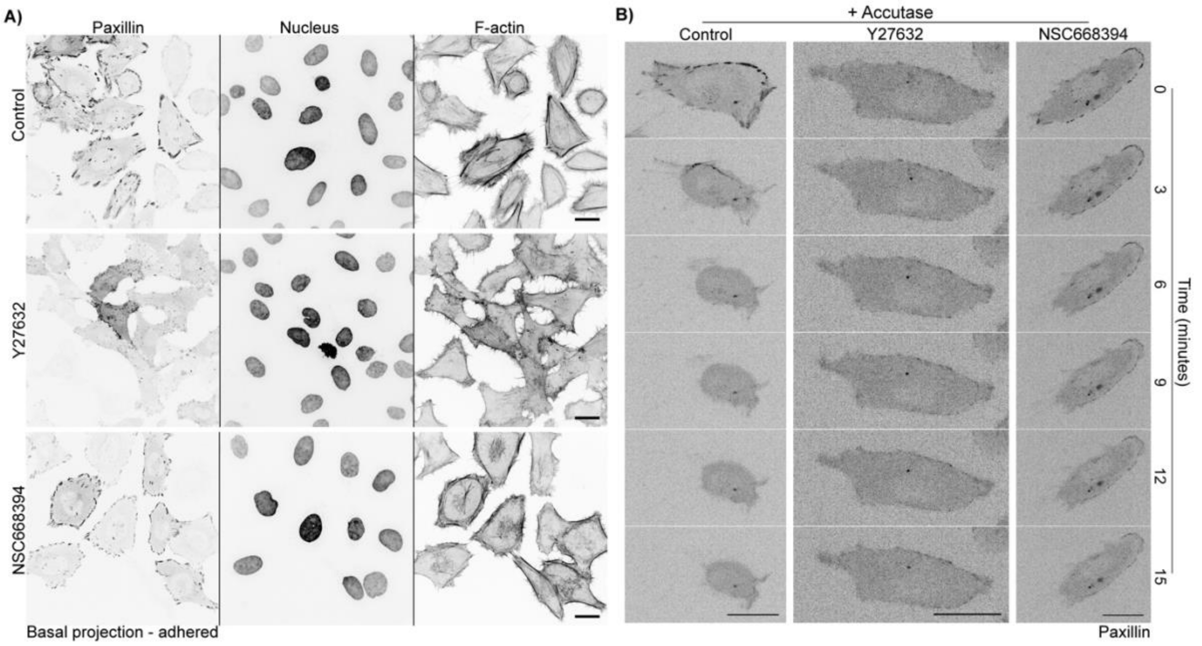

Supplementary Figure 3. Alteration to paxillin during ROCK or ezrin inhibition – related to Figure 2 and Figure 3.

A) Immunofluorescence images of control HeLa and after treatment with Y27632 (10µM, 20 minutes) or NSC668394 (250 µM, 3 hours), images in panel show from left to right: paxillin, cell nucleaus and F-actin, Scale bar = 20 µm; B) Time-lapse of control cells, or pre-treated with Y27632 (10µM, 20 minutes) or NSC668394 (250 µM, 3 hours), after addition of Accutase over 15 minutes, Grey – Paxillin, Scale bar = 20 µm.

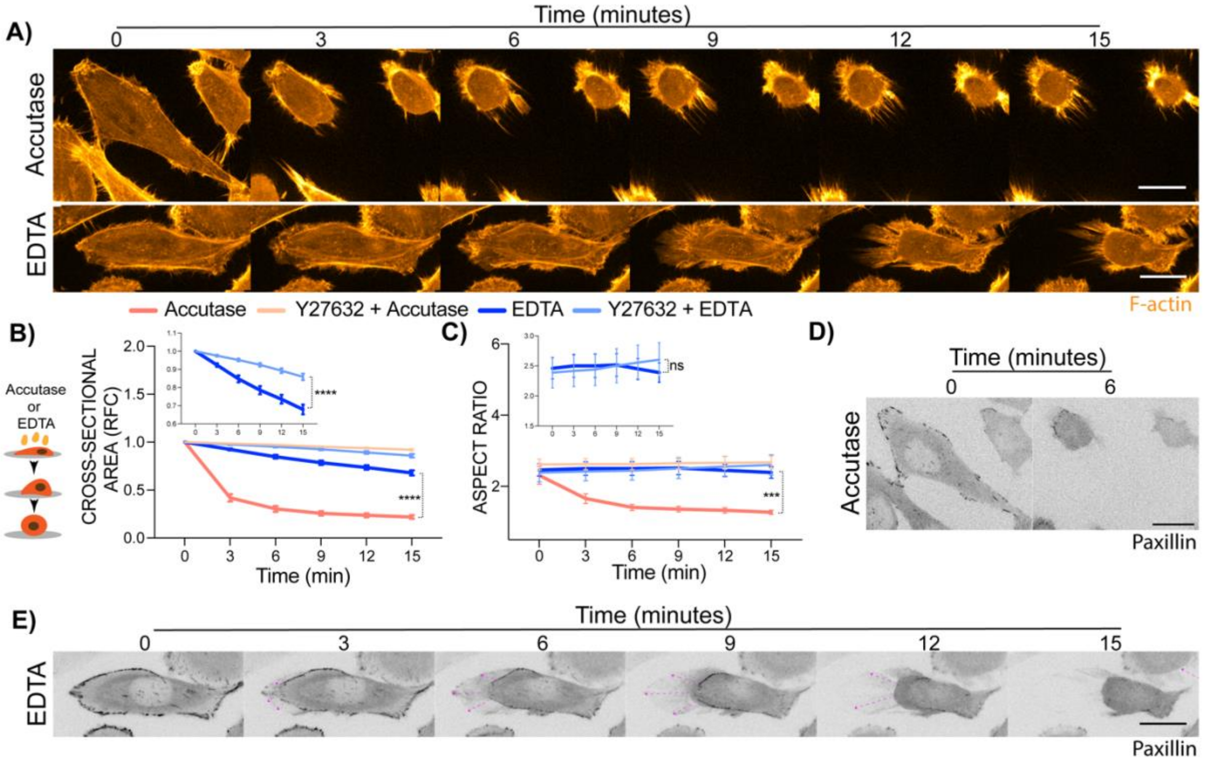

Supplementary Figure 4. Effect of EDTA on cell detachment in comparison to Accutase – related to Figure 1 and Figure 3.

A) Time-lapse images of HeLa cells detached either with Accutase or EDTA over 15 minutes, Orange – F-actin Scale bar = 20 μm; B-C) Measurement of Accutase (n=20) or EDTA-detached (n=25) HeLa cells over 15 minutes, either not pre-treated (control) or Y27362-pretreated cells: B) Cross—sectional area (relative fold change in area compared to time 0), C) Aspect ratio; Statistical difference: 2-way ANOVA with Tukey's multiple comparisons test, **p<0.01, ****p<0.001; D) Images of control cells detached with Accutase at 0 and 6 minutes of detachment, Grey - Paxillin; E) Time-lapse of control cells detached with EDTA over 15 minutes, Grey – Paxillin; Pink arrows to indicate not fully retracted cell body during detachment.

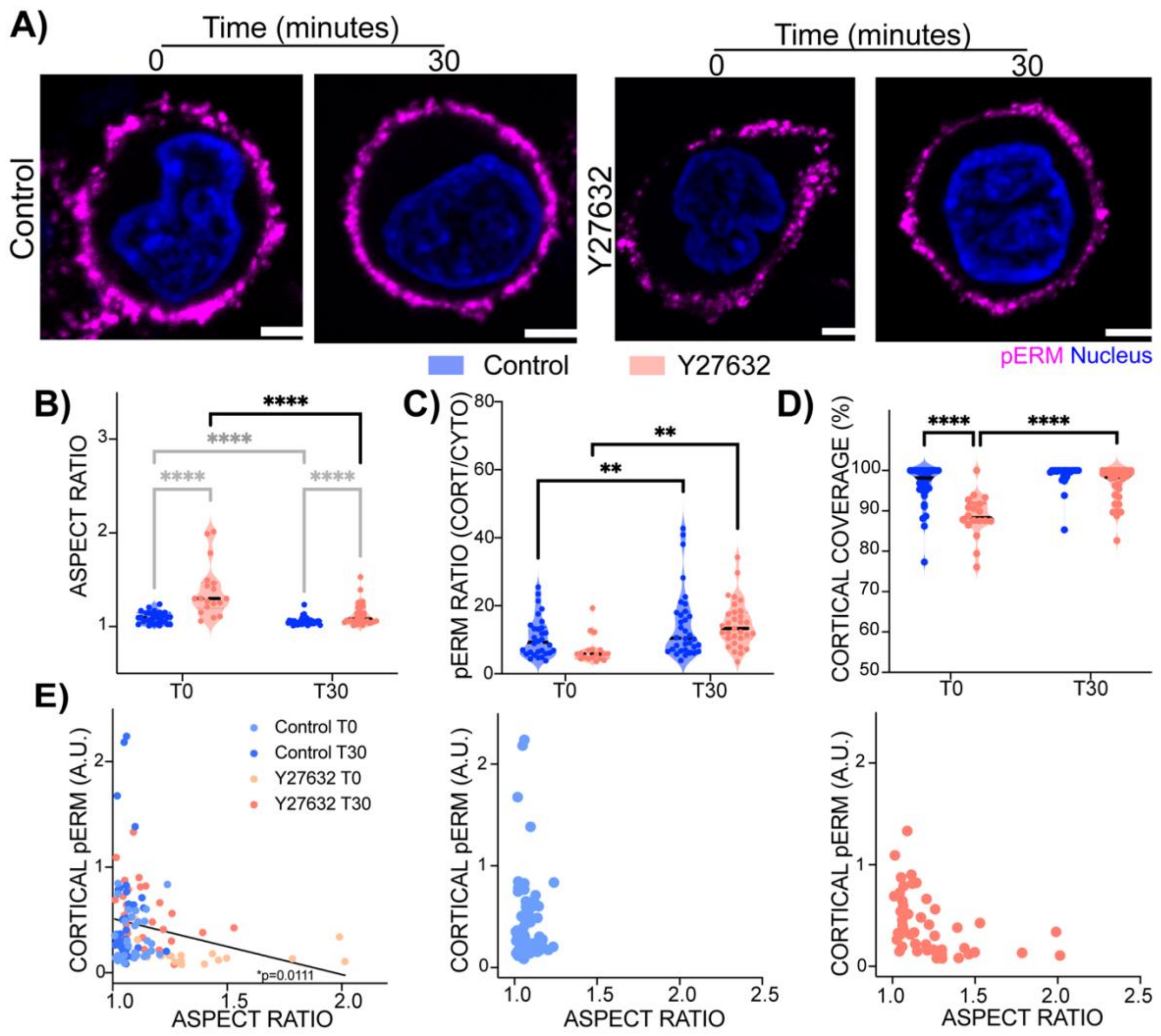

Supplementary Figure 5. Role of actin and ROCK signalling in rounding in suspension following detachment – related to Figure 3.

A) Immunofluorescence images of DMSO-treated (Control) or Y27632-treated HeLa cells after full detachment with Accutase and resuspension in cell culture media (T=0 minutes) and following further incubation in suspension (T=30 minutes), Magenta – pERM, Blue – Nucleus. Scale bar = 10 μm; B-E) Measurement of control or Y27632-treated cells in suspension from fixed immunofluorescence images: B) Aspect ratio; C) Ratio of the averaged cortical and cytoplasmic pERM; D) Percentage coverage of the cell cortex by pERM; Statistical difference: 2-way ANOVA with Tukey's multiple comparisons test, **$p<0.01$, ****$p<0.001$; E) Correlation between the cortical expression of pERM and aspect ratio for control and Y27632-treated cells, together with individual plots for control and Y27632-treated cells.
